# Radiofrequency ablation for stage <IIB non‐small cell lung cancer: Opportunities, challenges, and the road ahead

**DOI:** 10.1111/1759-7714.15114

**Published:** 2023-09-23

**Authors:** Qing Zhao, Jing Wang, Yi‐li Fu, Bin Hu

**Affiliations:** ^1^ Department of Thoracic Surgery, Beijing Chaoyang Hospital Capital Medical University Beijing China

**Keywords:** immunotherapy, non‐small cell lung cancer, prognosis, radiofrequency ablation, targeted drug

## Abstract

Pulmonary carcinoma represents the second common cancer for human race while its mortality rate ranked the first all over the world. Surgery remains the primary option for early‐stage non‐small cell lung cancer (NSCLC) in some surgical traditions. Nevertheless, only less than half of patients are operable subjected to the limited lung function and multiple primary/metastatic lesions. Recent improvements in minimally invasive surgical techniques have made the procedure accessible to more patients, but this percentage still does not exceed half. In recent years, radiofrequency ablation (RFA), one of the thermal ablation procedures, has gradually advanced in the treatment of lung cancer in addition to being utilized to treat breast and liver cancer. Several guidelines, including the American College of Chest Physicians (ACCP), include RFA as an option for some patients with NSCLC although the level of evidence is mostly limited to retrospective studies. In this review, we emphasize the use of the RFA technique in patients with early‐stage NSCLC and provide an overview of the RFA indication population, prognosis status, and complications. Meanwhile, the advantages and disadvantages of RFA proposed in existing studies are compared with surgical treatment and radiotherapy. Due to the high rate of gene mutation and immunocompetence in NSCLC, there are considerable challenges to clinical translation of combining targeted drugs or immunotherapy with RFA that the field has only recently begun to fully appreciate.

## Graphical abstract

Radiofrequency ablation (RFA) technology has begun to be widely used in lung cancer, but no unified standardized treatment guidelines have been formed worldwide, and there is a lack of clinical evidence for its application in patients with early‐stage lung cancer. This article focuses on the application of RFA in early‐stage non‐small cell lung cancer patients, including the indication population, patient prognosis, and complications, and also summarizes the advantages and disadvantages of surgery versus RFA based on the current research base. At the same time, this article focuses on the development of RFA in combination with targeted or immunotherapy to provide new research directions for the application of RFA in lung cancer.

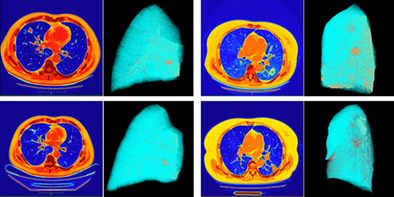

## INTRODUCTION

In recent years, with the application of low‐dose spiralcomputed tomography (CT) in clinical checkups, a large number of early lung cancers can be detected. Some data show that the detection rate of early lung cancer has increased by 20%–30%. In addition, the results of the latest global statistics show that lung cancer poses a major threat to human life with the second highest incidence rate and the first highest mortality rate in the world. Patients with NSCLC made up 85% of all lung cancer patients among them. Despite the fact that surgery is still the preferred course of action for patients with early‐stage non‐small cell lung cancer (NSCLC), the discipline of radiation oncology has had innumerable opportunities over the past 10 years to achieve high rates of local tumor control and cure.^1^ At the same time, more patients are unable to receive surgical treatment due to poor lung function brought on by underlying lung diseases that coexist, more challenging secondary surgery as a result of recurrence, as well as physical and psychological factors.^2^ For these individuals, CT‐guided percutaneous pulmonary radiofrequency ablation offers a more appropriate treatment option and NCCN guidelines recognize the use of this technique in high‐risk patients.^3^

**FIGURE 1:**
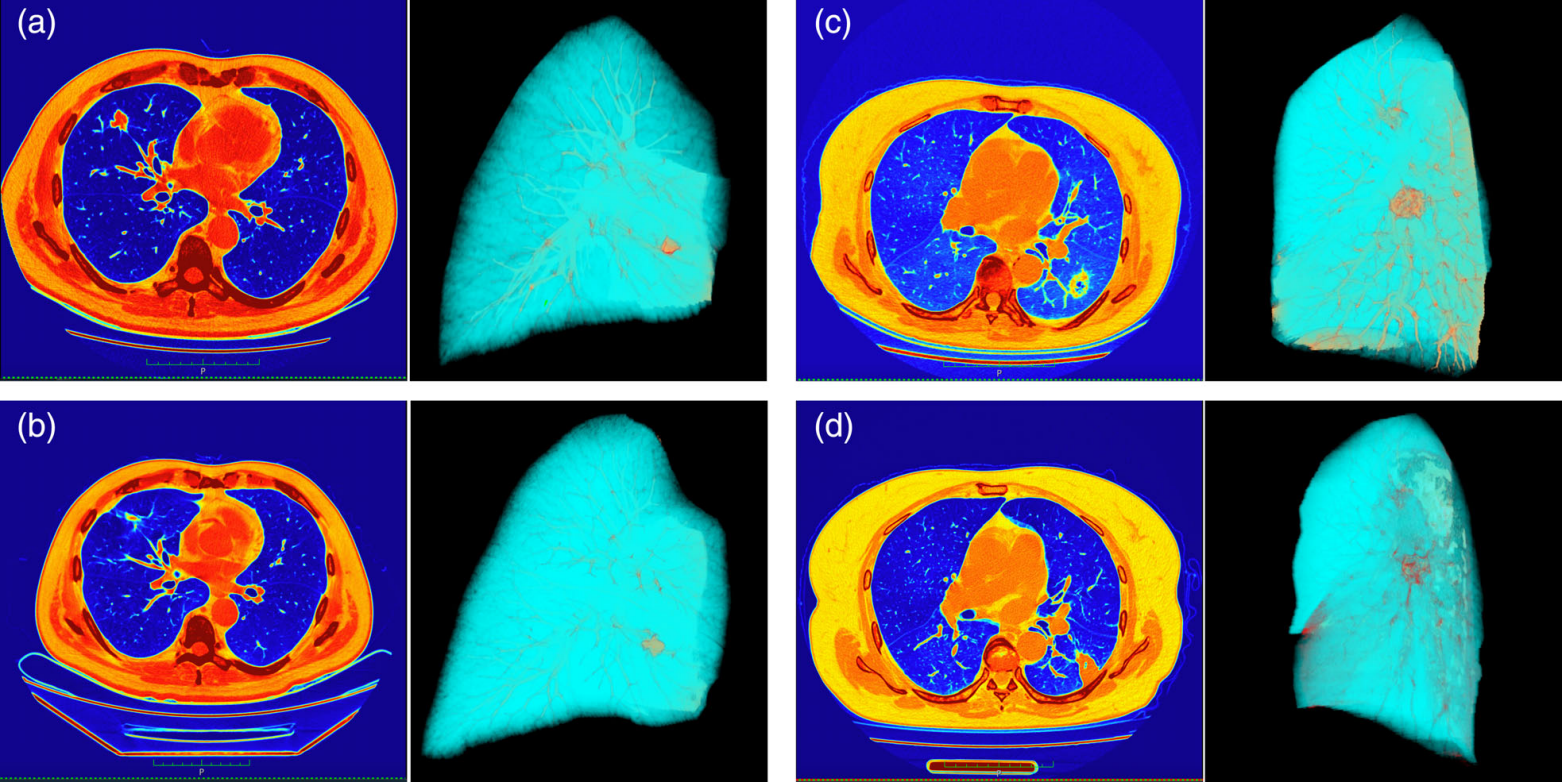
Changes in computed tomography imaging and alterations in thermodynamic three‐dimensional reconstruction maps before and after patients were treated with radiofrequency ablation (RFA). Imaging data for both patients were obtained from our team's non‐small cell lung cancer patient RFA treatment group follow‐up results. (a, c) Before RFA treatment. (b, d) After RFA treatment.

Radiofrequency ablation (RFA), as the most common type of thermal ablation technique, was first applied in 1989 to treat supraventricular tachycardia and other cardiac electrical conduction diseases when medication proved ineffective.^4^ RFA was first used to treat hepatocellular carcinoma in the middle of the 1990s, and its quick capacity to degenerate and necrotize tumor cells has given patients with inoperable tumors a new option.^5^ This technique has been gradually applied to thyroid nodules, bone and soft tissue tumors, breast cancer, and renal cell carcinoma.^6^, ^7^, ^8^, ^9^, ^10^ It was first reported to be used in the treatment of lung malignant tumors in 2000.^11^ In particular, the air‐containing nature of healthy lung tissue allows radiofrequency ablation to be better confined to tumors with low impedance, obtaining a necrotic border of cauterized similar to that of an R0 resection in surgery, and thus achieving complete ablation.^12^ The comparison of the thermodynamic images of the patients before and after RFA showed that although the volume of the tumor was increased due to the proliferation of the surrounding tissue after cauterization, the metabolism of the tumor tissue was significantly reduced (Figure 1a–d).

However, there are few clinical studies on RFA in early‐stage NSCLC patients, mostly focusing on retrospective comparisons of survival data with other single treatment modalities. This review summarizes the indication population, recurrence and prognosis, occurrence and causes of complications of RFA from the perspective of clinical application, and also compares RFA with surgery, radiotherapy, immunotherapy and targeted therapy to provide reference for clinicians' treatment choices.

## CURRENT APPLICATION STATUS FOR RFA IN EARLY‐STAGE NSCLC

The latest version of the Chinese expert consensus on thermal ablation therapy categorizes the indications for thermal ablation therapy as peripheral subsolid nodules and refusal of surgery for various reasons^13^; however, we believe that this categorization is unclear from the point of view of patient benefit. In this review we have divided patients treated with RFA for early‐stage NSCLC into two broad categories, patients who cannot tolerate or refuse surgery, and patients who may have better outcomes in RFA. The treatment of metastatic lung tumors often requires a multidisciplinary consultation compromise and is not included in the summary of this article at this time.

With the development and introduction of video‐assisted thoracoscopic surgery (VATS), thoracoscopic lobectomy and sublobectomy has always been the gold standard treatment for NSCLC in clinical guidelines. However, with the aging of the population, currently lung cancer patients are still mainly middle‐aged and elderly people.^14^ Some patients have more chronic diseases and postoperative complications may cause greater risks; some patients have low cardiac function and cannot tolerate surgical procedures under general anesthesia; and a small percentage of patients with long‐standing chronic obstructive pulmonary disease (COPD) have poor lung function and may not be able to ensure a normal quality of life after lobectomy.^15^, ^16^ In these cases, RFA and high‐dose radiotherapy become a more suitable option. Dupuy et al. found no significant change in lung function following RFA in patients with worse physical conditions.^17^ The 2021 guidelines for thermal ablation of primary lung tumors also state that “intolerance or subjective refusal of surgery and radiotherapy” in patients with NSCLC should be taken into consideration as category 2b evidence, strongly recommending RFA.^13^

In addition to inoperable patients, the feasibility of RFA should be fully considered in patients with multiple primary ground‐glass opacities (GGOs).^18^ The use of low‐dose thin‐layer CT in recent years has resulted in an annual rise in the detection rate of GGOs in young patients. Among them, multiple GGOs are defined as multiple independent primary foci that can present as various early pathological types such as Atypical adenomatous hyperplasia (AAH) and adenocarcinoma in situ (AIS). For such patients with multiple primary GGOs and not a centralized nodal distribution, the choice of surgical treatment is complicated by the combination of segmentectomy or wedge resection and the psychological stress of long‐term follow‐up. According to recent recommendations, the majority of numerous nodules on imaging that have a ground glass component are inert tumors that have a more favorable five‐year prognosis for the patients.^19^ In several retrospective studies of nodules with >50% GGO component treated with thermal ablation, patients were found to have a 100% disease‐specific survival (DSS) at both 3‐ and 5‐years postoperatively.^20^, ^21^ In contrast to solid nodules, it has also been proposed that nodules with a high GGO component include a large quantity of air and may be more likely to result in inadequate local control.^22^, ^23^ Therefore, a longer follow‐up time with a bigger clinical cohort comparison is still required to justify the utility of RFA in multiple nodules with GGO predominance.

## PROGNOSTIC INDICATORS FOR NSCLC PATIENTS AFTER RFA

### Relapse and progression

Local recurrence and progression of tumors after RFA treatment is one of the reasons limiting the widespread use of RFA. It is speculated that the reason for this phenomenon is that the heat sink effect caused by peritumoral vascular flow affects heat loss from the target tissue, resulting in incomplete necrosis.^24^ Blood can leak out due to the vascular damage brought on by RF, which can result in peripheral local recurrence.^17^ Toshihiro et al's follow‐up of GGO patients after RFA found that the rate of local tumor recurrence was as high as 17.6% after 18 months or more, much higher than that of surgical resection.^20^ The local recurrence‐free survival rates of T1a and T1b patients were not significantly different in the follow‐up of patients with early‐stage NSCLC after RFA, both approximating 60%.^17^ Although the ease and safety of RFA makes secondary or multiple radiofrequency achievable, the study by Hiraki et al. of local progression of secondary radiofrequency after RFA showed limited local control gains with repeated procedures for tumors with risk factors.^25^

The size of the tumor volume is the primary risk factor that has a significant impact on recurrence and progression after RFA. Although there is no uniformly certified guideline proposing a specific tumor diameter in relation to RFA prognosis, 3 cm appears to be a more important standard line. Several studies have confirmed that larger tumors have significantly higher recurrence rates after RFA, and Lee et al. discovered that in 30 patients who had pulmonary RFA, 100% complete necrosis was achieved in tumors less than 3 cm in size, compared to only 23% in tumors larger than 3 cm in size.^26^ In the updated data on prolonged follow‐up in patients with NSCLC by Ambrogi et al., the rate of imaging complete remission in tumors smaller than 3 cm was improved by 20% in tumors larger than 3 cm.^27^ The ACCP guidelines also include RFA as a treatment option for high‐risk patients with NSCLC less than 3 cm in size.^28^ However, some studies believe that this criterion can be suitably relaxed to 5 cm, particularly in individuals who are unable to undergo surgery. In the clinical cohort by Zhu et al., nodal foci larger than 5 cm were excluded from RFA surgery.^29^ In the study by Fernando et al., it was concluded that for patients with early‐stage NSCLC with high surgical risk, tumors up to 5 cm in diameter can still achieve near‐radiotherapy‐like results.^30^ These results suggest that RFA should be targeted at small tumors as much as possible and that a tumor diameter of 3 cm may be the borderline value for screening the population for universal indication of RFA. However, the boundary may be relaxed to 5 cm when the underlying health of the patient is taken into account.

The distance between the RFA cautery margin and the tumor margin is another parameter that is substantially linked with postoperative recurrence. The distance of the incision margin from the tumor is a critical consideration when developing a surgical plan to treat NSCLC. This metric is equally important when treating RFA. In a postoperative model of RFA in rabbit lungs, it was demonstrated that the peripheral edge of the RF ablation zone tended to mix necrotic cells with a small number of viable cells. If ablation is performed only to the tumor border, there is a substantial risk of recurrence due to residual viable cells.^31^ Due to hemorrhage and surrounding injured tissue, the volume of immediate ablation is apparently exaggerated under CT observation.^32^ Therefore, ablation that covers the tumor and extends beyond the tumor border is essential to reducing recurrence. It has been demonstrated that an outer layer of ground‐glass opaque strips encompassing the tumor with a thickness greater than or equal to 5 mm may be sufficient to ensure that the ablation is recurrence‐free, and the importance of a strip of at least 5 mm covering the tumor margins has been confirmed by Yamamoto et al.^33^ The absence of this opaque strip may correspond to the site of postoperative recurrence.^34^ When the strip area was four times the size of the tumor area, the complete ablation rate climbed to 96%, according to Baere et al.^35^ Therefore, ensuring the largest possible ablation area to achieve complete ablation is one of the factors influencing the reduction of recurrence and progression.

### Overall survival (OS) and DSS

In the several clinical studies available, we found that 1‐year OS in patients undergoing RFA was generally quite impressive, ranging from 83% to 98%, whereas 5‐year OS floated between 22.5% and 55.7%. In one major study that enrolled 5268 NSCLC patients over an 11‐year period, the 1‐year OS of the RFA‐treated group improved to 84.2% compared to 62.8% in the untreated control group.^36^ However, there may be some inaccuracies in investigating the OS of individuals treated with RFA. Most of the patients included in the study were NSCLC patients who were inoperable on their own underlying conditions, and other comorbidities may have increased the risk of mortality from causes other than tumor. The 5‐year OS of patients receiving RFA was determined to be 25% in the prospective single‐center research by Ambrogi et al., but the DSS increased to 40% when other disease factors were excluded.^27^ In the RAPTURE study, the 2‐year OS after RFA was 48% in 33 NSCLC patients, while the 2‐year DSS reached 92%.^37^ The research by Simon et al. further supported this view. When comorbidity in patients was quantified using the Charlson Comorbidity Index (CCI), patients with low‐scoring NSCLC had considerably higher median survival than patients with high‐scoring NSCLC.^38^ Therefore, we believe that DSS should be emphasized more than OS when assessing the prognosis of NSCLC patients treated with RFA. We searched PubMed with the keywords “early‐stage NSCLC” and “RFA,” included cohort studies with clear prognostic survival rates from the year 2006 onwards. The summary of previous studies reporting survival outcomes is shown in Table 1.

**TABLE 1:** Studies in the literature reporting survival outcomes of patients with primary lung cancer who underwent radiofrequency ablation (RFA) included in this review.

References	Number of patients	Tumor types	Mode of intervention	OS/CSS
Grieco, et al. (2006)^77^	41	Inoperable clinical stage I/II NSCLC	Radiofrequency ablation and microwave ablation, followed by RT.	The OS was 97.6% at 6 months, 86.8% at 1 year, 70.4% at 2 years, and 57.1% at 3 years.
Simon, et al. (2007)^39^	153	Stage I NSCLC	CT‐guided radiofrequency ablation	The 1‐, 2‐, 3‐, 4‐, and 5‐year OS for stage I NSCLC were 78%, 57%, 36%, 27%, and 27%
Hiraki, et al. (2007)^78^	20	Inoperable clinical stage I NSCLC	Radiofrequency ablation	The OS and CSS were 90% and 100% at 1 year, 84% and 93% at 2 years, and 74% and 83% at 3 years
Lanuti, et al. (2008)^79^	31	Inoperable clinical stage I NSCLC	Radiofrequency ablation	The 2‐ and 4‐year OS were 78% and 47%
Lencioni, et al. (2008)^37^	33	Inoperable early stage NSCLC	Radiofrequency ablation	The 1‐, 2‐year OS in the NSCLC were 70% and 48%. The 1‐, 2‐CSS in the NSCLC were 92% and 73%
Zemlyak, et al. (2010)^40^	64	Stage I NSCLC unfit for standard resection	Sublobar resections (SLR), radiofrequency ablation (RFA), and percutaneous cryoablation therapy (PCT)	The 3‐year OS for the SLR, RFA, and PCT groups was 87.1%, 87.5%, and 77%. The 3‐year cancer‐specific and cancer‐free survival for SLR, RFA, and PCT groups was 90.6% and 60.8% versus 87.5% and 50% versus 90.2% and 45.6%
Ambrogi, et al. (2011)^27^	57	Inoperable clinical stage I NSCLC	Radiofrequency ablation	The CSS was 89% at 1 year, 59% at 3 years, and 40% at 5 years.
Kim et al. (2011)^80^	22	Stage I NSCLC	Radiofrequency ablation, surgery	The 1‐, 2‐, and 5‐year OS of 88%, 50%, and 25% in the RFA group vs. 93%, 77%, and 67% in surgery group
Kodama et al. (2012)^81^	44	Recurrent NSCLC	Radiofrequency ablation	The 1‐, 3‐, and 5‐year OS were 97.7, 72.9, and 55.7%
Dupuy, et al. (2015)^17^	54	Inoperable clinical stage IA NSCLC	CT‐guided radiofrequency ablation	The 1‐, 2‐year OS were 86.3%, 69.8%
Lam, et al. (2018)^82^	335	Early stage NSCLC	Radiofrequency ablation, stereotactic body radiotherapy	The 1‐, 3‐, and 5‐year OS of 85.5%, 54.3%, and 31.9% in the SBRT group vs. 89.3%, 52.7%, and 27.1% in the RFA group
Uhlig, et al. (2018)^83^	28 834	Stage I NSCLC	Thermal ablation, stereotactic radiation therapy	The 1‐, 2‐, 3‐, 5‐year OS between TA and SRT were 85.4% vs. 86.3%, 65.2% vs. 64.5%, 47.8% vs. 45.9% and 24.6% vs. 26.1%
Li, et al. (2020)^36^	189	Inoperable stage IA NSCLC	Radiofrequency ablation	The 1‐, 3‐, and 5‐year OS in the unmatched RFA and NT groups were 84.2%, 49.0%, and 29.4% vs. 62.8%, 31.1%, and 17.1%
Lu, et al. (2022)^2^	18	Inoperable clinical stage I NSCLC	Radiofrequency ablation	The 1‐, 2‐, and 3‐year OS were 92.2%, 81.5%, and 54.3%

Abbreviations: CSS, cancer specific survival; NSCLC, non‐small cell lung cancer; NT, no treatment; OS, overall survival; PCT, percutaneous cryoablation therapy; SBRT, stereotactic body radiotherapy; SRT, sterotactic radiotherapy.

## COMPLICATIONS AFTER RFA TREATMENT

### Pneumothorax

Pneumothorax is the most common postoperative complication of RFA. In a Japanese retrospective study for a 10‐year period, the incidence of pneumothorax after RFA was 52%. The probability of pneumothorax in different studies averaged around 30% and fluctuated widely.^25^ This occurrence might be brought on by the inconsistent diagnosis of pneumothorax in various areas, where some studies classified any visible pneumothorax on imaging as a problem while others concentrated on the existence of pneumothorax‐related clinical symptoms.^29^ However, one point of view that can be harmonized is that most postoperative pneumothorax after RFA does not require much particular management, with chest drainage rate ranging from 1%–9.8% or even lower, and the existence of this complication has not been demonstrated to have a significant impact on patients' long‐term survival.^39^, ^41^, ^42^

Risk factors for the development of pneumothorax may include old age, previous emphysema, number of tumors greater than 2, tumors farther away from the chest wall, more lung tissue for the electrodes to pass through, and routes through the interlobular spaces.^43^ Among these, the number of tumors greater than 2 and the length of lung tissue crossed by the electrodes were found to be extremely significant independent risk factors.^29^, ^44^ Taken together, more pleural damage and lung tissue damage are the two main reasons for the appearance of pneumothorax after RFA.

### Pleural effusion

Pleural effusion after RFA occurs in 4% to 21% of patients.^29^, ^36^, ^45^ Similar to pneumothorax, most patients with pleural effusion after RFA do not require treatment.^46^ The process of RFA employs the biological effect of heat to cause tissue destruction. Needle electrodes penetrate alternating current deep into the lung tissue, generating ionic agitation that has a thermobiological effect. Elevated temperatures at the lesion can cause coagulative necrosis of tumor cells. However, the heat transfer to the pleura may induce pleurisy, which promotes the formation of pleural effusion.^47^

### Hemorrhage

RFA may cause vascular injury when tumors are located near important blood vessels in the lung due to a lack of localization guidance or heat transfer during penetration maneuvers. The clinical incidence of RFA combined with hemorrhage ranges from 1% to 18%.^48^ Most bleeding is self‐limiting, but serious complications such as hemothorax and hemoptysis are possible. Acute hemorrhage can rapidly reduce oxygen saturation and cause death in patients with poor respiratory reserve function.^49^ Pulmonary artery pseudoaneurysm is a much rarer complication of bleeding. Over a 6‐year period study in Japan, only one patient with pseudoaneurysm‐related hemoptysis was observed after 538 RFA procedures. It is speculated that this may be due to simultaneous necrosis of the lung tumor and the adjacent pulmonary artery wall during RFA, resulting in the formation of a fistula between the cavity within the tumor and the pulmonary artery.^50^ Therefore, the risk of such complications should be fully considered when treating tumors near pulmonary artery branches.

Small lesion size, lesion location at the base, electrode penetration through a large amount of lung tissue, electrode paths through the pulmonary vasculature, and the use of multipolar ablation are operational risk factors for complications associated with intraoperative and postoperative bleeding during RFA.^49^ The intercostal arteries should also be prioritized during initial access to the chest cavity. If the patient has a pre‐existing condition such as coagulopathy or high pulmonary artery pressure, it may be difficult to suppress bleeding once it occurs. As a result, we believe that thorough preoperative examination and RFA pathway planning can reduce the likelihood of such complications.

### Rare complications

Pneumonia, pleurisy, bronchopleural fistula, lung abscess, delayed varicoceles in the cavity, gas embolism, rib or vertebral injury, pericardial effusion, cardiac arrhythmia, myocardial infarction, and other complications are possible after RFA.^25^, ^51^ Although the probability of these complications is extremely low, they frequently require immediate management or follow‐up. When patients develop relevant clinical symptoms following RFA, we cannot rule out the possibility of these complications developing.

## DEVELOPMENT IN COMPARISON WITH SURGICAL TREATMENT

Surgery is the most widely accepted treatment option for patients with NSCLC and has a significantly better prognosis than other modalities. However, only a small number of patients can undergo standard lobectomy according to the guidelines, and more elderly patients are unable to tolerate surgery or subjectively resist lobectomy due to their poor underlying conditions. RFA has the advantages of a simple operation, a short treatment time, high safety, and little impairment of lung function, which undoubtedly provides a new option for these patients to enhance their prognosis and quality of life.

The phenomenon that the recurrence rate after RFA is higher than that of surgical treatment has been confirmed in many studies. In the Z4033 study, the first multicenter prospective evaluation of the prognosis of RFA, a local recurrence rate of approximately 40% at 2 years after RFA was found to be significantly higher than the 7.7% local recurrence rate at 3 years after sublobar resection.^17^ However, a recent meta‐analysis comparing RFA to surgery in patients with stage I NSCLC found that, while survival rates were lower in RFA patients than in surgery patients, the underlying conditions of RFA patients were significantly worse, suggesting that the treatment effect of RFA may have been underestimated in many studies.^52^ After propensity score matching for the patient condition, Kwan et al. demonstrated that long‐term survival in the RFA group was not significantly different from that in the surgical group.^53^ Primary thoracic surgery frequently results in significant thoracic adhesions that make secondary surgery impossible. RFA, on the other hand, does not interfere with the operation of secondary or multiple ablations after surgery. The superimposed number of treatments has also been shown to significantly improve local control,^25^ so that local recurrence in the short term may not affect long‐term patient survival. Furthermore, in terms of hospitalization days and treatment costs, RFA outperforms surgical treatment.^54^ We believe that RFA is an appropriate treatment option in patients with NSCLC who are unable to undergo surgery for various reasons.

Despite the large number of clinical studies that have used RFA as a second option for patients who cannot tolerate surgery or have intermediate to advanced NSCLC, some studies have affirmed the good performance of RFA in patients with stage I NSCLC. In a study of 64 patients with stage I NSCLC who were treated with either surgery or RFA, Zemlyak et al. found that the 3‐year OS in patients with RFA was not different from that of the surgical group.^40^ Safi et al. compared stage I NSCLC patients who underwent sublobar resection with RFA patients and found that the surgical group had a better local control rate, but there was no difference in OS resulting from the two treatment modalities.^55^ Kim et al. made the same observation after matching the clinical characteristics of stage I NSCLC patients under both treatment modalities.^56^ However, the number of patients included in the above studies was too small and the lack of prospective cohort studies was insufficient to affirm the efficacy of RFA in patients with early‐stage NSCLC. Still, the results of these studies open up new ideas as to whether RFA can be used as a treatment modality of equal choice to surgery in patients with stage I NSCLC, a direction that clearly deserves further validation.

RFA also has an obvious drawback compared to surgical treatment, which is the inability to obtain specifics of tumor pathology and lymph nodes. In traditional surgical treatment, postoperative tumor pathology results and metastasis of lymph nodes are extremely important information that can be used to assess the TNM stage of a patient's tumor. The ACCP guidelines suggest that systematic lymph node dissection is not an optional operation in surgery for patients with early‐stage NSCLC, and that postoperative platinum chemotherapy should be considered for patients with stage IIA who are in good physical condition.^57^ At the same time, the application of targeted drugs is also strongly dependent on the genetic mutation status of the tumor tissue. During RFA, preoperative puncture to obtain pathological biopsies does not guarantee that enough tissue will be obtained for pathological and genetic testing, and the possibility of obtaining biopsies is even more lost after cauterization. Therefore, most of these patients could not be further combined with other treatments as after surgical treatment. We believe that the implications of this problem should be considered comprehensively when choosing RFA as the primary treatment modality.

## DEVELOPMENT IN COMPARISON WITH RADIOTHERAPY

The use of radiotherapy in NSCLC patients has been clinically validated in a large number of cases, among which, stereotactic radiotherapy (SBRT) is recommended by several guidelines as the standard of care for patients with inoperable early‐stage NSCLC. SBRT treats tumors with highly focused high‐dose radiation, and some studies have shown that the local control rate and survival rate of patients significantly increase with higher SBRT doses.^58^ The clinical prospective trial RTOG0236 reported favorable results in nonsurgical patients with early‐stage NSCLC, with 3‐ and 5‐year OS reaching 55.8% and 40%, respectively.^59^

The efficacy and patient benefit of RFA and SBRT as concurrent options for nonsurgical patients are controversial. The retrospective study by Li et al. of 6195 patients with stage IA nonsurgical NSCLC found no significant difference in OS between the RFA and SBRT groups.^60^ The meta‐analysis by Zhang et al., which included 105 studies, concluded that RFA and SBRT were similar only in short‐term OS, but SBRT was superior in long‐term OS.^61^ Meanwhile, SBRT seems to have better results in terms of local control of tumors. Bi et al. found that SBRT patients had better local control than RFA patients at 1, 2, 3, and 5 years after surgery, after correcting for differences in patient general condition and tumor size.^62^ However, it has also been suggested that the definition of local recurrence can change depending on the chosen treatment modality, and without pathological evidence, it is difficult to determine whether SBRT causes complete local tumor destruction.^55^, ^63^ Therefore, the existing clinical studies comparing postoperative tumor recurrence in SBRT and RFA patients may not be rigorous.

The combination of RFA with SBRT seems to be proven feasible in recent years. Dupuy et al. reported the outcome of 24 patients with the combination at 2‐year follow‐up, suggesting a better local control than RFA treatment alone.^64^ In patients presenting with recurrence after radiotherapy, Cheng et al. found that thermal ablation prolonged the local control of recurrent tumors and may be an effective salvage treatment.^65^ There are also controversies in theoretical studies of combination therapy. Schoellnast et al. suggested that pulmonary fibrosis after radiotherapy may impair the accuracy of RFA localization and affect the RFA effect.^66^ However, Dupuy et al. suggested that hypoxic cells in the tumor center may be the reason for the poor radiotherapy effect, and RFA destroys the tissue in hypoxic area by thermal ablation, allowing radiation to act again and favor the radiotherapy effect.^64^

Several cohort studies have suggested the advantages of this combination application; however, the recommended use of RFA in combination with SBRT has not appeared in any of the numerous guidelines and multicenter follow‐ups for NSCLC patients. We believe that the indication for the combination may emerge in some patients after adequate multidisciplinary evaluation in large hospitals, but currently this combination is not an option that can be widely recommended.

## COMBINATION OF RFA WITH TARGETED THERAPY AND IMMUNOTHERAPY

Finding ways to boost patients' immune responses has become a new direction in tumor treatment in recent years, and immunotherapy based on targeted drugs has significantly improved NSCLC patients' prognosis. Among these, the PD‐L1/PD‐1 axis between T cells and tumor cells promotes tumor growth by activating inhibitory signaling pathways. RFA was discovered to have two primary functions in the study: activating the immune system and increasing tumor‐targeted receptors, providing evidence for combining RFA with targeted and immunotherapy.

Targeted drug therapy has a significant prognostic enhancing effect for tumor patients with specific genetic loci as oncogene research advances. Among them, the *EGFR* gene, which is more commonly mutated in NSCLC patients, has been targeted with successive generations of newer EGFR tyrosine kinase inhibitors (TKIs). Several clinical cohorts have confirmed the significant advantages offered by EGFR‐TKI therapy over standard chemotherapy and its importance in patients with the presence of *EGFR* mutations. However, in many NSCLC patients who can be treated with existing TKI agents, targeted agents are not a choice that can be taken with a high degree of confidence. Secondary drug resistance can be caused by a number of drug‐resistant mutations, including T790M mutation, MET amplification, PIK3CA mutation, ERBB2 amplification, epithelial‐mesenchymal transition, and pathological type transformation. Multiple clinical studies have confirmed that the median time to drug resistance in patients responding to TKI therapy is 9–13 months.

For these patients who have progressed again after the application of targeted drug therapy, discontinuation and switching to chemotherapy with cytotoxic agents is the conventional option available. However, the application of thermal ablation therapy as a local treatment not only prolongs the survival time of patients but also offers them the possibility of reapplying targeted drugs. Several clinical studies have confirmed that local thermal ablation therapy improves patient prognosis after drug resistance progression. In patients with oligometastatic TKI resistance, Xu et al. discovered a significant survival benefit with the addition of local ablation therapy.^67^ A clinical study by Ni et al. found that when MWA was administered to patients who developed EGFR‐TKI progression, reintroduction of the same TKI drug significantly prolonged patient OS and PFS.^68^

Some experimental studies have provided possible answers to the question of why thermal ablation improves the drug resistance status of patients with targeted drugs. When patients are treated with local thermal ablation, some of the resistant tissues become necrotic through the thermal effect. The rest of the tumor tissue is still sensitive to treatment and can be treated with the original targeted drugs.^69^ T90M mutation is the most common in EGFR‐positive patients, and osimertinib (OSI, also known as AZD9291), a third‐generation EGFR‐TKI drug, is highly selective for T90M mutation carriers. According to one study, patients who have the T90M mutation deleted may be resistant to OSI. As seen in cellular experiments, heat effects increased T90M mutation levels in cells and cell proliferation levels were altered by exposure to heat damage.^70^ As a result, RFA in combination with OSI may represent a novel treatment option for patients with OSI‐resistant NSCLC.

At the same time, the combination of RFA and immunotherapy has shown promising results because RFA generates a large amount of debris tissue in situ, including tumor cell fragments that can activate antitumor immune responses and damage‐associated molecular patterns (DAMPS) that cause intrinsic immune responses. Tumor cell fragments contribute to antigen‐presenting cell (APC) maturation and dendritic cell infiltration thereby promoting antitumor immunity. These fragmented tissues act as new antigens, stimulating an increase in the T cell‐based immune response while decreasing the number of Treg cells that regulate immunosuppression.^71^, ^72^ In post‐thermal ablation studies of various cancers such as liver and pancreatic cancer, it was found that increased infiltration of CD4+ and CD8+ T cells and decreased proportion of Treg cells in the immune environment after RFA attenuated the immunosuppressive effect in the tumor microenvironment.^73^ However, the effect of these RFA‐induced immune responses was not long‐lasting, and combined immunotherapy was required to better enhance patient prognosis. The question of how to combine RFA with targeted immunotherapy has become a new direction of exploration.

Despite the lack of clinical evidence in NSCLC patients, local RFA has been shown to increase PD‐L1 expression in distant tumors in a variety of cancers, suggesting that patients with tumors lacking PD‐L1 receptors may benefit from immunotherapy.^74^, ^75^ Tumor foci treated with RFA were found to be significantly more responsive to the PD‐L1 monoclonal antibody atelectilizumab than those not treated with RFA in a comparative study of two squamous cancer metastases in a patient with NSCLC.^76^ This case confirms the synergistic effect of RFA in combination with immunotherapy in NSCLC patients, but it still needs to be validated in a larger patient cohort. Several clinical cohort studies on the use of thermal ablation in combination with immunotherapy in lung cancer patients are already underway, and the results may broaden the indication population for existing immunotherapy and provide additional evidence to support the dissemination of RFA in combination with immunotherapy.

## CONCLUSION

In conclusion, we have summarized the application of RFA in NSCLC patients. Cancer, as a disease requiring comprehensive management, must strike an acceptable balance for patients. Because of its improved safety and operability, RFA provides a therapeutic outlet for NSCLC patients who cannot tolerate surgery. Meanwhile, the good therapeutic effect of RFA provides a simpler modality option for GGO patients. Although the rate of local recurrence after RFA is higher than that of surgical treatment, the long‐term prognosis is still promising, and its application in early‐stage NSCLC patients is worthy of discussion. The development of targeted and immunotherapy therapy provides a new avenue for postoperative combination therapy for RFA, which needs to be validated in further large‐scale cohorts.

## AUTHOR CONTRIBUTIONS

Conceptualization—Qing Zhao, Jing Wang. Literature research—Xin Li, Ying Ji, Yan Zhao, Yi Liu. Manuscript editing—Yi‐li Fu. Supervision—Bin Hu. All authors contributed to the article and approved the submitted version.

## FUNDING INFORMATION

Beijing Institute of Respiratory Disease Reform and Development Project, Grant/Award Number: Ggyfz202316; Beijing Chaoyang hospital Golden Seed Research Project, Grant/Award Number: CYJZ202206.

## CONFLICT OF INTEREST STATEMENT

The authors declare that the research was conducted in the absence of any commercial or financial relationships that could be construed as a potential conflict of interest.
